# Sexual behaviours and associated factors among students at Bahir Dar University: a cross sectional study

**DOI:** 10.1186/1742-4755-11-84

**Published:** 2014-12-06

**Authors:** Wondemagegn Mulu, Mulat Yimer, Bayeh Abera

**Affiliations:** Bahir Dar University, College of Medicine and Health Sciences, Department of Medical Microbiology, Immunology and Parasitology, Bahir Dar, Ethiopia

**Keywords:** Sexual behaviours, University students, Associated factors, Bahir Dar

## Abstract

**Background:**

Sexual behaviour is the core of sexuality matters in adolescents and youths. Their modest or dynamic behaviour vulnerable them to risky sexual behaviours. In Ethiopia, there is scarcity of multicentered representative data on sexual behaviours in students to have a national picture at higher education. This study therefore conducted to assess sexual behaviours and associated factors at Bahir Dar University, Ethiopia.

**Methods:**

A cross sectional study was conducted among Bahir Dar University students from December to February 2013. Multistage sampling and self administered questionnaires were employed. Descriptive statistics such as frequency and mean were used to describe the study participants in relation to relevant variables. Multivariate analysis was carried for those variables that had a p-value of ≤ 0.2 in the bivariate analysis to identify the predictor variables.

**Results:**

Of the 817 study participants, 297 (36.4%) students had ever had sex. The mean age at first sexual practice was 18.6 years. Unprotected sex, having multiple sex partners, sex with commercial sex workers and sex for the exchange of money was practiced by 184 (62%), 126 (42.7%), 22 (7.4%) and 12 (4%) of sexually active students, respectively. The proportion of attending night clubs and watching porn videos was 130 (15.8%) and 534 (65.4%), respectively. Male respondents had significant positive association with watching porn videos (AOR = 4.8, CI = 3.49 - 6.54) and attending night clubs (AOR = 3.9, CI = 2.3 – 6.7). Watching porn videos, attending night clubs, khat chewing and taking alcohol frequently were significantly associated for ever had sex and having multiple sexual partners. Khat chewing practice (AOR = 8.5, CI =1.31 - 55.5) and attending night clubs (AOR = 4.6, CI = 1.8 - 11.77) had statistical significant association with the purpose of sexual intercourse for the sake of money and for having sex with commercial sex workers, respectively.

**Conclusions:**

Significant number of students had different risky sexual behaviours. Substance use, attending night clubs and watching porno video were predictor factors for practicing different sexual behaviours. Therefore, preventive intervention programmes should be strengthened, effectively implemented and monitored both in the earlier school and in the universities.

## Introduction

In adolescents and young people risky sexual behaviour has been recognized as an important health, social and demographic concern in the developing world[1]. Adolescent and youth are vulnerable to many health problems. Because they often have multiple sexual relationships and inconsistent use of condoms[2]. Young men may have their first sexual experiences with prostitutes, while young females may have their first sexual experiences with older men, both of which increase the chance of getting sexually transmitted infections (STIs) including Human Immunodeficiency Virus (HIV)[1, 2]. Substance abuse exposes the users to risky sexual behaviours such as having unprotected sex which can have economic, social, physical, psychological, and health problems[2, 3].

University students are in the youth age category and are exposed to risky sexual behaviours such as unprotected sexual intercourse leading to HIV, other STIs and unwanted pregnancies[4–6]. Female youths are prone to unwanted pregnancies that lead unsafe abortions, severe illness, infertility and death[3, 7].

Young people aged 10–24 years constitute around 1.8 billion and represent 27% of the world’s population[7]. Studies noted that as they are in the youth age category, their modest or dynamic behaviour vulnerable them to risky sexual behaviours[7, 8]. Sexually transmitted diseases like HIV/AIDS and other reproductive health (RH) problems are the greatest threats to the well-being of adolescents and youth[7, 9].

Globally, one-third of the 340 million new STIs cases occur per year in people under 25 years of age. Each year, more than one in every 20 adolescents contracts a curable STI. Studies reported that more than half of all new HIV infections occur in people between the ages of 15 and 24 years[7, 10].

In Ethiopia, young people (aged 15–24) represented one of the country’s largest groups, comprising about 35% of the population[11]. To enhance the sexual and reproductive health and well-being of the young’s population, Ethiopia had a national strategies and activities. Some of the strategies are delivery of all youth RH-related interventions and policies by gender, age, marital status, and residence; addressing the immediate and long-term RH needs of young people; and strengthening multicultural partnerships to respond to young women’s heightened vulnerability to sexual violence and non-consensual sex[7, 12]. Some of the activities are creating awareness of sexual health, providing youth friendly services, increasing human resource capacity, explore new opportunities and expand multtisectorial coordination[7, 12]. However, most related interventions targets the general public as a result it do not directly respond to higher education institution students need and expectation, making actual coverage of behavioural and biomedical interventions extremely low[13]. Therefore, sexual behaviour among adolescents and the youth is still a major issue in Ethiopia[11].

Previous studies conducted in other universities of Ethiopia showed a 26.9% to 34.2% of students ever had sexual intercourse. Of them, 45.2% had more than one sexual partner and 59.4% had first sex at high school. Moreover, the mean age at first sexual practice was 17.9 year and 4.4% of participants had sex with commercial sex workers[4, 14–16]. Inadditon, different scholars also reported that different factors are responsible for the sexual behaviours of adolescents. Among those, use of alcohol and chewing khat are the common factors[4, 17–19].

Although Ethiopia is in a concerted effort to enhance the sexual behaviour of youths using different strategy, activities and policies at the national level, the epidemic still continues to grow steadily in the country especially in educational setting claiming the lives of the most productive segments of the Ethiopian society that can lead to high social and economic costs, both immediately and in the years ahead. Moreover, dynamicity of adolescent’s behaviour; it is assumed that student’s sexual behaviour varies interms of locality, civilization, urbanization and socio cultural context of the societies. Specifically, Bahir Dar University is located in areas where there is high flow of tourists, comfortable pensions and night clubs that will expose the students to be engaged in different sexual risk behaviours. However, with the above problems, there is paucity of multicentered data representing the sexual behaviours of students in higher level institution at national level and also among Bahir Dar University students.Therefore, the purpose of this study was to assess the sexual behaviours and associated factors among students of Bahir Dar University, Ethiopia.

## Methods

### Study design, period and area

A cross-sectional study was conducted among students in Bahir Dar University (BDU) from December to February 2013. BDU is a public higher educational institution established in 2000[20]. The University is located in Bahir Dar town 567 kilometres Northwest of Addis Ababa. It offers a wide range of higher education programs both at undergraduate and graduate levels[20]. BDU is now among the largest Universities in the Federal Democratic Republic of Ethiopia, with more than 35,000 students in its 57 undergraduate and 39 graduate programmes. At the time of the study, it has four campuses (Main campus, Poly campus, Zenzelma and Yibab campus) in Bahir Dar which had about 20,000 full time undergraduate students[20]. BDU has five student clinics. They are engaged in youth friendly services. At the time of data collection, information and counselling on sexual and reproductive health issues, promotion of healthy sexual behaviours through various methods including peer education, family planning information, counselling and method including emergency contraceptive methods and condom promotion and provision and abortion linkage service provided in youth friendly service. Currently each clinic has three nurses trained about youth friendly services[20, 21].

### Study population

All full time undergraduate students attending at Bahir Dar University during the study period.

### Inclusion criteria

Full time undergraduate students ranging from year I to year V were included in the study.

### Exclusion criteria

Post-graduate, extension, summer, advance standing and distance learning students were excluded during data collection.

### Sample size and sampling technique

#### Sample size determination

The sample size was determined using single population proportion formula considering the following assumptions: P = 50% (The expected proportion of ever had sex among the students), 95% confidence level and marginal error of 5%.

The formula for calculating the sample size is:

$$\begin{array}{ll} \;\frac{\left(z\alpha /2\right)2\;p\left(1-p\right)}{d^{2}} & ={\left(1.96\right)}^{2}\left(0.5\;\times \;0.5\right)/{\left(0.05\right)}^{2}\\ =384,n=384 \end{array}$$

Assuming 10% non-response rate, design effect 2, the sample size was: n =384 × 2 + 10% = 768+ 76.7 = 848. The final sample size was 848. However, only 817 BDU students completed the questionnaire adequately.

### Sampling procedure

Multistage sampling was used. To assure the representativeness of the data, the sample size was proportionally allocated to each college proportional to their number of students. Simple random sampling technique was used to select the departments from each year of study in the seven colleges. Finally the study participants were selected using systematic random sampling technique.

### Variables of the study

#### Dependent variable

Sexual behaviours such as ever had sex, unprotected sex, having multiple sex partners, sex for the exchange of money and sex with commercial sex workers.

### Independent (explanatory) variables

Socio-demographic variables such as age, sex, year of study, religion, ethncity, marital status and place of residence, alcoholism, khat chewing, attending night clubs and watching porn video.

### Operational definition

#### Unprotected sex

Having sex without condom during their sexual experience.

#### Protected sex

Using condom during each and every sexual intercourse.

#### Ever had sex

Penile - vaginal sexual intercourse during each and every sexual intercourse.

### Data collection procedures

A structured and self-administered questionnaire, which was partly adopted from Ethiopia Demographic and Health Survey (EDHS), Behavioural Surveillance Survey (BSS) and other relevant sources were used to collect the data[22, 23]. All questionnaires were completed individually in the student clinic.

### Data quality control issues

To maintain the quality of data, training was given to data collectors and supervisors on how to approach and select the study participants, on the objective of the study and the content of the questionnaire. Structured and self administered questionnaire was used. Questionnaire was pre-tested by taking 85 students from the university other than the actual study participant’s. The questionnaire was first prepared in English and translated in to Amharic language for appropriateness and easiness. The Amharic version was again translated back to English to check for consistency of meaning.

### Data analysis

The data was analysed using SPSS version 20. Descriptive statistics such as frequencies and mean were used to describe the study participants in relation to relevant variables. Most of the variables were fitted to the bivariate logistic regression. Then all variables having a p value of ≤0.2 in the bivariate analysis were further entered in to logistic regression model. In the multivariate analysis, backward step wise logistic regression techniques were fitted and confounding and multicolinearity were controlled. Variables having p value < 0.05 in the multivariate analysis were taken as significant predictors. Crude and adjusted odds ratios with their 95% confidence intervals were calculated. The Hosmer and Lemshow gardens-of-fit test was used to assess whether the necessary assumptions for the application of multiple logistic regression were fulfilled and p- value > 0.05 was considered a good fit.

### Ethical clearance

Ethical clearance was obtained from Ethical review committee of Bahir Dar University, College of Medicine and Health Sciences. A formal approval was secured from Bahir Dar University and informed consent was obtained from the respondents before proceeding to the data collection. Confidentiality of the result was also maintained.

## Results

### Socio-demographic characterstics

A total of 817 full time undergraduate students with a response rate of 96.7% participated in the study. Of these, 545 (66.7%) were males. The mean age of the respondents was 21 years ranged from 18 to 30 years. Majority 618 (75.6%) of them were between 20–24 years. In ethncity, 466 (57.1%) were from Amhara and 147 (18%) were Oromo. Regards to religion, 624 (76.4%) of the respondents were Orthodox Christian follower. In this study, 704 (86.4%) were unmarried. Five hundred ten (62.4%) of the study participants were either year one or two students. About, 802 (98.2%) of the respondents live in the campus dormitory (Table 1).

**Table 1:** **Socio-demographic variables, ever had sex, multiple sex partners and unprotected sex among Bahir Dar University students, 2013**

Variables	Ever had sex (N = 817)			Ever had multiple sex partners (N = 297)		Unprotected sex (N = 297)	
	Yes N (%)	No N (%)	Total N (%)	Yes N (%)	No N (%)	Yes N (%)	No N (%)
**Sex**							
Male	229 (42)	316 (58)	545 (66.7)	110 (48.5)	16 (23.5)	138 (60.3)	91(39.7)
Female	68 (25)	204 (78)	372 (33.3)	117 (51.5)	52 (76.5)	46 (67.6)	22 (32.4)
**Age**							
18-20	35 (21.)	131 (79)	166 (20.3)	16 (45.7)	19 (54.3)	22 (62.9)	13 (37.1)
20-24	239 (38.7)	379 (61.3)	618 (75.6)	98 (41.2)	140 (58.8)	145 (60.7)	94 (39.3)
>24	23 (70)	10 (30)	33 (4)	12 (54.5)	10 (45.5)	17 (51.5)	6 (48.5)
**Religion**							
Orthodox	236 (37.8)	388 (62.2)	624 (76.4)	92 (39.3)	142 (60.7)	145 (61.7)	90 (38.3)
Muslim	22 (26.8)	60 (73.2)	82 (10)	8 (36.4)	14 (63.6)	14 (60.9)	9 (39.1)
Protestant	29 (32.2)	61 (67.8)	90 (11)	18 (14.3)	3 (1.8)	21 (72.4)	8 (27.6)
Other	10 (47.6)	11 (52.4)	21 (2.6)	8 (40)	12 (60)	4 (40)	6 (60)
**Year of study**							
Year one	63 (24.8)	191 (75.2)	254 (31.1)	23 (37.1)	39 (62.9)	40 (64.5)	22 (35.5)
Year two	98 (38.3)	158 (61.7)	256 (31.3)	43 (43.4)	56 (56.6)	65 (61.9)	35 (38.1)
Year three	98 (49.7)	99 (50.3)	197 (24.1)	44 (45.8)	52 (54.2)	57 (59.4)	39 (40.6)
Year four	24 (36.9)	41 (63.1)	65 (8)	9 (37.5)	15 (62.5)	16 (66.7)	8(33.3)
Year five	14 (31.1)	31 (68.9)	45 (5.5)	7 (50)	7 (50)	6(40)	9 ( 60)
**Place of residence**							
In the University	291 (36.3)	511 (63.7)	802 (98.2)	524 (65.3)	122 (96.8)	180 (62.1)	110 (37.9)
Out of campus	6 (40)	9 (60)	15 (1.8)	10 (66.7)	167 (98.8)	4 (57.1)	3 (42.9)
**Marital status**							
Single	241 (34.2)	463 (65.8)	704 (86.2)	109 (45.6)	130 (54.4)	141 (58.8)	99 (41.3)
Married	47 (46.5)	54 (53.5)	101 (12.4)	15 (31.9)	32 (68.1)	36 (75)	12 (25)
Divorced	5 (71.4)	2 (28.6)	7 (0.9)	1 (20)	4 (80)	5 (100)	0 (100)
Other	4 (80)	1 (20)	5 (0.6)	1 (25)	3 (75)	2 (50)	2 (50)
**Ethncity**							
Amhara	153 (32.8)	313 (67.2)	466 (57.1)	52 (34.2)	100 (65.8)	104 (67.5)	50 (32.5)
Oromo	60 (40.8)	87 (59.2)	147 (18)	35 (59.3)	24 (40.7)	31 (52.5)	28 (47.5)
Tigray	38 (45.8)	45 (54.2)	83 (10.2)	18 (47.4)	20 (52.6)	21 (55.3)	17 (44.7)
Others	46 (38.3)	74 (61.7)	120 (14.7)	21 (45.7)	25 (54.3)	28 (60.9)	18 (39.1)
**Total**	**297 (36.4)**	**520 (63.6)**	**817 (100)**	**127 (42.8)**	**170 (57.2)**	**184 (70)**	**113 (30)**

### Sexual practice

The overall proportion of ever had sexual practice was 297 (36.4%). In the present study, ever had multiple sex partners was 126 (42.7%) of the sexually active students. Having multiple sexual partners was 110 (48.5%) and 16 (23.5%) in males and females, respectively. Regards to condom use, 113 (38%) of the sexually active respondents had consistently used condom during sex. Watching porn videos was noted in 534 (65.4%) of respondents. The highest proportion 421 (77.2%) was found in males (Table 1).

Sexual intercourse for the exchange of money was found in 12 (4%) of the sexually active respondents (Table 2). The mean age at first sexual practice was 18.6 years. Seventy two (24.3%) of the respondents initiated sexual activity before the age of 18 years. Moreover, among those respondents who ever had sex, 174 (58.6%) had started sex during secondary school. However, 33 (11.1%) had first sex during elementary school (Table 3).

**Table 2:** **Socio-demographic variables, watching porn videos, attending night clubs and sex for the exchange of money among Bahir Dar University students, 2013**

Variables	Watching porn video (N = 817)		Sex for the exchange of Money (N = 297)		Attending night clubs (N = 817)	
	Yes N (%)	No N (%)	Yes N (%)	No N (%)	Yes N (%)	No N (%)
**Sex**						
Male	421 (77.2)	124 (22.8)	7 (3.1)	221 (96.6)	112 (20.6)	433 (79.4)
Female	113 (41.5)	159 (76.5)	5 (7.2)	64 (92.8)	18 (6.6)	254 (93.4)
**Age**						
18-20	93 (56)	73 (44)	1 (2.9)	34 (97.1)	23 (13.9)	143 (86.1)
20-24	414 (67)	204 (33)	10 (4.2)	229 (95.8)	99 (16)	519 (84)
>24	27 (81.8)	6 (18.2)	1 (4.3)	22 (95.7)	8 (24.2)	25 (75.8)
**Religion**						
Orthodox	413 (66.2)	211 (33.8)	8 (3.4)	227 (96.6)	103 (16.5)	521 (83.5)
Muslim	48 (55.8)	38 (44.2)	1 (4.3)	21 (95.7)	9 (11)	73 (89)
Protestant	6 (75)	2 (25)	0 (0)	27 (93.1)	10 (11.1)	80 (88.9)
Other	67 (65)	36 (35)	1 (9.1)	10 (90.9)	8 (38.1)	13 (61.9)
**Year of study**						
Year one	147 (57.9)	107 (42.1)	4 (6.3)	59 (93.7)	26 (10.2)	228 (89.8)
Year two	171 (66.8)	85 (33.2)	4 (3.9)	96 (96.1)	45(17.6)	211 (82.4)
Year three	147 (74.6)	50 (25.4)	4 (4.1)	92 (95.5)	46 (23.4)	151 (76.6)
Year four	42 (64.6)	23 (35.4)	0 (0)	24 (100)	9 (13.8)	56 (86.2)
Year five	27 (60)	18 (40)	0 (0)	14 (100)	4 (8.9)	41 (91.1)
**Place of residence**						
In the university	524 (65.3)	278 (34.7)	11 (3.8)	280 (96.2)	127 (15.8)	675 (84.2)
Out of campus	10 (66.7)	5 (33.3)	1 (16.7)	5 (83.3)	3 (20)	12 (80)
**Marital status**						
Single	458 (65.1)	246 (34.9)	11 (4.6)	230 (95.4)	17 (16.8)	84 (83.2)
Married	69 (68.3)	32 (31.7)	1 (2.1)	46 (97.9)	110 (15.6)	594 (84.4)
Divorced	3 (42.9)	4 (57.1)	0 (0)	5 (100)	1 (14.3)	6 (85.7)
Other	4 (80)	1 (20)	0 (0)	4 (100)	1 (40)	3 (60)
**Ethncity**						
Amhara	288 (61.8)	178 (38.2)	6 (3.9)	147 (96.1)	62 (13.3)	404 (86.7)
Oromo	106 (72.1)	41 (27.9)	3 (5)	57 (95)	28 (19)	119 (81)
Tigray	57 (68.7)	26 (31.3)	2 (5.3)	36 (94.7)	22 (26.5)	61 (73.5)
Others	83 (69.2)	37 (30.8)	1 (2.2)	45 (97.8)	17 (14.2)	103 (85.8)
**Total**	**534 (65.4)**	**283 (34.6)**	**12 (4.0)**	**285 (96)**	**130 (15.9)**	**687 (84.1)**

**Table 3:** **Other sexual and related behaviours of students at Bahir Dar University in relation to male and female, 2013**

Variables	Male N (%)	Female N (%)	Total N (%)
**Age at first sex (N = 297)**			
10-17	56 (24.7)	16 (23.2)	72 (24.3)
18-19	106 (46.7)	30 (43.5)	136 (45.9)
>20	65 (28.6)	23 (33.3)	89 (29.7)
**Starting time of sexual intercourse (N = 297)**			
Elementary school	24 (10.5)	9 (13)	33 (11.1)
High/preparatory school	142 (62.3)	32 (46.4)	174 (58.6)
In university	58 (25.4)	25 (36.2)	83 (27.9)
Other	4 (1.8)	3 (4.3)	7 (2.4)
**Reason for having multiple sexual partners (N = 297)**			
To get partner with good sexual pleasure	40 (35.4)	1 (7.7)	41 (32.6)
Beautiful/handsome of partner	21 (18.6)	2 (7.7)	22 (17.5)
To get matured sexual partner	9 (8)	3 (23.1)	12 (9.5)
Effect of long term relation ship	27 (24)	2 (15.4)	29 (23)
Seeking for money	5 (4.4)	1 (7.7)	6 (4.8)
Other	11 (9.7)	5 (35.7)	16 (12.7)
**Had sex with older individuals (N = 297)**			
Yes	52 (22.7)	12 (17.6)	64 (21.5)
No	177 (77.3)	56 (82.4)	233 (78.5)
**Sex after watching porn videos (N = 297)**			
Yes	64 (27.9)	9 (13.2)	73 (24.6)
No	165 (72.1)	59 (86.8)	224 (75.4)
**Sex after drinking alcohol (N = 297)**			
Yes	88 (38.4)	14 (20.6)	102 (34.3)
No	141 (61.6)	54 (79.4)	195 (65.7)
**Had sex with commercial sex workers (N = 297)**			
Yes	22 (9.6)	0 (0)	22 (7.4)
No	207 (90.4)	68 (100)	275 (92.6)
**Frequency of condom use (N = 297)**			
Regularly	91 (39.7)	22 (32.4)	113 (38)
Frequently	71 (31)	11 (16.2)	82 (27.6)
Some times	15 (6.6)	10 (4.4)	25 (8.4)
Never	52 (22.7)	25 (10.9)	77 (25.9)
**Reason for not using condom (N = 184)**			
Difficult to get condom	19 (13.7)	2 (4.4)	21 (11.4)
In love with a partner	27 (19.4)	16 (35.6)	43 (23.4)
Condom reduce sexual satisfaction	56 (40.3)	11 (24.4)	67 (36.4)
My partner is free for HIV	36 (25.9)	13 (28.9)	49 (26.6)
For money	1 (0.7)	3 (6.7)	4 (2.2)
**Reason for not practising sex until study period (N = 520)**			
Fear of HIV/other STIs	46 (14.6)	10 (4.9)	56 (10.8)
Fear of family	7 (2.2)	1 (0.5)	8 (1.5)
Need to wait until marriage	192 (60.8)	171 (83.8)	363 (69.8)
To prevent unwanted pregnancy	5 (1.6)	9 (4.4)	14 (2.7)
Need to wait until marriage + Fear of HIV	52 (16.5)	9 (4.4)	61 (11.7)
Has no specific reason	14 (4.4)	4 (2)	18 (3.5)

Regarding the reason for ever had multiple sexual partners, seeking sexual pleasure and the effect of long term relationship was the major reason in males and females, respectively. On the other hand, among respondents who were not using condom consistently, 67(36.4%) reported that condom decreases sexual pleasure. Moreover, condom use reduces sexual pleasure was the leading reason among males while in love with a partner was the major reason among females for unprotected sex (Table 3). More importantly, need to wait until marriage, 363 (69.8%) was the major reason for not initiating sexual intercourse and other reasons are listed in Table 3.

Ever had sex with commercial sex workers has been reported by 27 (7.4%) of respondents. Sixty four (21.5%) of sexually active students had an experience of sexual intercourse with older individuals. Engaging in sexual intercourse after watching porn videos, drinking alchol and chewing khat were noted in 73 (24.6%), 102 (34.3%) and 51 (17.2%) of those students who ever had sexual intercourse respectively (Table 3).

### Multivariate analysis on sexual behaviours

On multivariate analysis, age difference had significant association with ever had sex and watching porn videos. Those respondents with age of 20–24 years (AOR = 9.5, CI = 3.75 - 23.85) and >24 years (AOR =3.65, CI = 1.7 - 7. 8) respectively were 10 and 3.6 times more likely to ever had sex. Those, respondents in the age group of > 24 years were 3 times more likely to watch pornographic material than students with age group of <20 years (AOR = 3.0, CI = 1.05 - 8.39). Likewise, sex difference showed significant association with history of watching porn videos, attending night clubs and ever had sex for the exchange of money. Male respondents were 4.1 times to ever had watch porno videos compared to female respondents (AOR = 4.1, CI = 2.88 - 5.75). However, female respondents were almost 3.7 times to practice sexual intercourse for the exchange money compared to male respondents (AOR = 3.7, CI = 1.04 – 13.2) (Table 4). Furthermore, more males were night club attendants than females (AOR = 2.3, CI = 1.25 -3.43) (Table 5).

**Table 4:** **Bivariate and multivariate analysis of factors associated with ever had sex, having multiple sex partners, and sex for the exchange of money among students of Bahir Dar University, 2013**

Variables		Ever had sex		Having multiple sexual partners		Sex for the exchange of money	
		COR (95 % CI)	AOR (95 % CI)	COR (95 % CI)	AOR (95 % CI)	COR (95 % CI)	AOR (95 % CI)
**Sex**							
	Male	2.2 (1.6 - 3.0)**	0.9 (0.6 - 1.3)	3.1 (1.7 - 5.7)**	1.4 (0.7 – 3.0)	2.4 (0.8 - 7.9)	3.7 (1.1 - 13.2)**
	female	^1^	^1^	^1^	^1^	^1^	^1^
**Age**							
	18-20	^1^	^1^		^1^		
	21-24	8.6 (3.75 - 19.78)**	9.5 (3.8 - 23.9)**	**─**	**─**	**─**	**─**
	>24	3.65 (1.7 - 7.8)**	3.6 (1.6 - 8.3)**	**─**	**─**	**─**	**─**
**Year of study**							
	Year I	^1^	^1^		**─**	**─**	**─**
	Year II	0.53 (0.4 - 0.8)**	0.8 (0.5 - 1.3)	**─**	**─**	**─**	**─**
	Year III	0.3 (0.2 - 0.5)**	0.6 (0.4 - 1.0)	**─**	**─**	**─**	**─**
	Year IV	0.6 (0.3 - 1.0)	0.9 (0.5 - 1.8)	**─**	**─**	**─**	**─**
	Year V	0.7 (0.4 - 1.5)	1.1 (0.5 - 2.4)	**─**	**─**	**─**	**─**
**Ethncity**							
	Amhara	**─**	**─**	0.6 (0.3 - 1.2)	0.9 (0.4 - 2.2)	**─**	**─**
	Oromo	**─**	**─**	1.7 (0.8 - 3.8)	1.6 (0.6 - 4.3)	**─**	**─**
	Tigray	**─**	**─**	1.1 (0.5 - 2.5)	1.3 (0.5 - 3.9)	**─**	**─**
	Other	**─**	**─**	^1^	^1^	**─**	**─**
**Religion**							
	Orthodox	1.5 (0.6 - 3.6)	0.9 (0.3 - 3.2)	0.2 (0.0 - 0.8)	0.4 (0.1 - 2.7)	**─**	**─**
	Muslim	2.5 (0.9 - 6.6)	1.7 (0.5 - 6.1)	0.1 (0.2 - 0.8)	0.3 (0.0 - 2.5)	**─**	**─**
	Protestant	1.9 (0.7 - 5.0)	1.1 (0.3 - 4.1)	0.4 (0.1 - 2.3)	1.3 (0.2 - 9.6)	**─**	**─**
	Other	^1^	^1^	^1^	^1^		
**Attending night clubs**							
	Yes	12.9 (7.9 - 21.1)**	7.4 (4.2 - 12.9)**	6.8 (3.9 - 11.5)**	3.1 (1.7 - 5.7)**	**─**	**─**
	No	^1^	^1^	^1^	^1^	**─**	**─**
**Watching porn video**							
	Yes	3.3 (2.4 - 4.7)**	1.8 (1.2 - 2.6)**	5.7 (2.6 - 12.5)**	2.8(1.1 - 6.9)**	**─**	**─**
	No	^1^	^1^	^1^	^1^	**─**	**─**
**Alcoholism**							
	Never	^1^	^1^	^1^	^1^	^1^	^1^
	Always	4.3 (3.1 - 5.8)**	1.9 (1.4 - 2.8)**	9.47 (2.6 - 34.7)**	4.4 (0.9 - 20.8)	0.1(0.0 - 0.8)	2.3 (0.4 - 13.7)
	Sometime	2.4 (1.1 - 4.1)**	2.2 (0.9 - 5.5)	6.3 (3.5 - 11.2)**	2.8 (1.4 - 5.6)**	0.4 (0.1 - 1.8)	0.04 (0.0 - 1.5)
**Chewing khat**							
	Never	^1^	^1^	^1^	^1^	^1^	^1^
	Sometimes	12 (4.1 - 35.6)**	3.3 (1.0 - 11.2)**	5.2 (2.9 - 9.0)**	2.3 (1.2 - 4.5)**	0.3 (0.1 - 0.9)**	8.5 (1.3 - 55.5)**
	Always	2.6 (0.8 - 8.2)	1.7 (0.5 - 6.2)	9.9 (3.2 - 31.0)**	2.8 (1.4 - 5.7)**	0.2 (0.0 - 1.1)	1.8 (0.3 - 10.1)

Key: COR: crude odds ratio, AOR: adjusted odds ratio, CI: Confidence interval, −**-**: those variables who had p-value >0.2 in bivariate analysis, ^1^: Reference category, **Significant association.

**Table 5:** **Bivariate and Multivariate analysis of factors associated with watching porn video, attending night clubs and sex with commercial sex workers among students of Bahir Dar University, 2013**

Variables		Watching porn videos		Attending night clubs		Sex with commercial sex workers	
		COR (95% CI)	AOR (95% CI)	COR (95% CI)	AOR (95% CI)	COR (95% CI)	AOR (95% CI)
**Sex**							
	Male	4.8 (3.5 - 6.5)**	4.1 (2.9 - 5.8)**	3.9 (2.3 - 6.7)**	2.3 (1.3 - 4.3)**	NA	NA
	female	^1^	^1^	^1^	^1^		
**Age**							
	18 – 20	^1^	^1^				
	20 – 24	3.5 (1.4 - 9.0)**	1.3 (0.9 - 2.0)	_	_	_	_
	> 24	2.2 (0.9 - 5.5)	3.0 (1.1 - 8.4)**	_	_	_	_
**Year of study**							
	Year I	^1^	^1^	^1^	^1^		
	Year II	0.7 (0.5 - 0.9)	1.2 (0.8 - 1.8)	1.2 (0.4 - 3.5)	2.3 (0.6 - 9.2)	**─**	**─**
	Year III	0.5(0.3 - 0.7)	1.6 (0.9 - 2.6)	2.2 (0.8 - 6.4)	3.4 (0.9 - 13.5)	**─**	**─**
	Year IV	0.8 (0.4 - 1.3)	1.2 (0.6 - 2.5)	3.1 (1.1 - 9.2)**	3.7 (0.9 - 14.4)	**─**	**─**
	Year V	0.9 (0.5 - 1.8)	1.3 (0.6 - 2.8)	1.7 (0.5 - 5.7)	2.8 (0.6 - 13.5)	**─**	**─**
**Ethncity**							
	Amhara	1.4 (0.9 - 2.1)	0.7 (0.4 - 1.2)	0.9 (0.5 - 1.7)	1.8 (0.8 - 3.7)	**─**	**─**
	Oromo	0.9 (0.5 - 1.5)	1.3 (0.7 - 2.4)	1.4 (0.7 - 2.8)	1.95 (0.8 - 4.5)	**─**	**─**
	Tigray	1.02 (0.6 - 1.9)	1.2 (0.6 - 2.3)	2.2 (1.1 - 4.4)**	5.6 (2.2 - 14.2)**	**─**	**─**
	Other	^1^	^1^	^1^	^1^	**─**	**─**
**Religion**							
	Orthodox	**─**	**─**	1	^1^		
	Muslim	**─**	**─**	0.6 (0.3 - 1.3)	0.73 (0.3 - 1.8)	**─**	**─**
	Protestant	**─**	**─**	0.6 (0.3 -1.3)	0.73 (0.3 - 1.9)	**─**	**─**
	Other	**─**	**─**	3.1 (1.3 - 7.7)**	4.0 (1.0 - 16.1)	**─**	**─**
**Chewing**							
**khat**	Never	^1^	^1^	^1^	^1^	^1^	^1^
	Some times	4.1 (2.4 - 6.9)**	2.0 (1.1 - 3.6)**	9.8 (6.3 - 15.4)**	6.48 (3.9 - 10.9)**	0.3 (0.1 - 0.9)**	0.54(0.2 - 1.5)
	Always	1.1 (0)	1.1 (0)	27.2 (10.8 - 68.3)	18.7 (6.4 - 53.9)**	0.3 (0.1 - 1.1)	0.38 (0.1 - 2.2)
**Alcoholism**							
	Never	^1^	^1^	^1^	^1^	^1^	^1^
	Always	1.2 (0.6 - 2.4)	0.61(0.3 - 1. 4)	9.3 (3.6 - 24)**	3.3 (1.1 - 10.1)**	0.3 (0.0 - 3.3)	0.8 (0.1 - 9.9)
	Sometimes	6.4 (4.4 - 9.4)**	4.78 (3.2 - 7.2)**	15.8 (8.9 - 27.7)**	9.5 (5.2 - 17.5)**	0.2 (0.1 - 0.9)**	0.6 (0.1 - 2.3)
**Attending**							
**night clubs**	Yes	4.2 (0.3 - 7.2)	0.9 (0.5 - 1.8)	NA	NA	4.2 (1.7 - 10.8)**	4.6 (1.8 - 1.8)**
	No	^1^	^1^			^1^	^1^

Key: COR: Crude odds ratio, AOR: adjusted odds ratio, CI: Confidence interval, −-, those variables who had p-value of > 0.2 in the bivariate analysis, NA: Not applicable, ^1^: Reference category, **Significant association.

The proportion of ever had sex did not vary significantly by year of study and religion. Likewise, having multiple sexual partners did not vary significantly by sex, religion and ethncity (Table 4). Proportion of unprotected sex did not vary significantly by age, sex, residence, year of study, ethncity, religion and other explanatory variables.

Students who watched porn videos were 1.8 times more likely to had sex compared to non users (AOR = 1.8, CI = 1.19 – 2.59). Likewise, respondents who watched porn videos were 2.8 times more likely to had multiple sexual partners compared to those who did not watch porn videos (AOR = 2.8, CI = 1.12 - 6.9). Night club attendants were 7 times more likely to had sexual practice (AOR = 7.4, CI = 4.23 -12.92) (Table 4). Similarly, attending night clubs was also the statistically significant associated factor for commencing sex with commercial sex workers (AOR = 4.6, CI = 1.8- 11.77) (Table 5).

Drinking alcohol regularly (AOR = 1.9, CI = 1.35 – 2.83) was also an associated factor for ever had sexual intercourse. The proportion of having multiple sexual partners were more among sometimes alcohol drunkers than non drunkers (AOR =2.8, CI = 1.4 – 5.6) (Table 4). For attending night clubs, drinking alcohol irregularly (AOR = 9.5, CI = 5.2 - 17.5) and regularly (AOR = 3.3, CI = 1.1 - 10.1) were also statistically significant (Table 5).

Had multiple sexual partners was also 2.8 times more likely among khat chewers compared to non chewers (AOR = 2.8, CI = 1.4 – 5.69). Chewing khat practice also significantly associated factor in engaging sexual intercourse for money (AOR = 8.5, CI = 1.31 – 55.5) (Table 4). Furthermore, chewing khat regularly (AOR = 1.98, CI = 1.08 - 3.64) and drinking alcohol (AOR = 4.78, CI = 3.17-7.20) were statistically significant associated factors for watching porn videos (Table 5).

## Discussion

In this study 36.4% of the students ever had sexual intercourse. This result is comparable to a study conducted in Nigeria (34%)[24]. However, this proportion was higher than findings of BSS-II (9.9%)[23], studies of other universities (26.9% to 34.2%), Ethiopia[4, 14–16] and a study conducted in Indian university (5% for female and 15% for male students)[25]. In contrast, it is the lowest compared to other studies in Africa. For instance, 49% to 59% of ever had sex in College and University students was reported in South Africa[26] and Uganda[27]. The possible explanation for the disparity in the proportion of sexual intercourse among adolescents of different studies could be due to different in traditional cultural back ground, sociodemographic characterstics, as well as difference in knowledge, attitude and practice towards HIV/AIDS.

Age at first sexual practice is an important indicator of exposure to the risk of unwanted pregnancy and STIs. The mean age at first sexual practice (18.6 years) for both males and females in this study is comparable to the reports of EDHS (18.2 years)[22], other universities in Ethiopia[14–16] and students of Madagascar (18.4 years)[26]. In contrast, the mean age of first sex was a bit higher than findings of Jimma University (17.7 years)[4] and Gomo Gofa (17 years)[28]. Moreover, more than half (58.6%) of sexually active students had first sex during secondary school. This conforms with studies of other Universities in Ethiopia ranged from 58.5% to 75.2%[4, 14–16]. This might indicate that early sexual imitation problem is the issue not only at university level, but also at high school and elementary level. Therefore, secondary school students should be targeted with preventive interventions as youth to discourage premature initiation of sexual activity. The result of multiple logistic regression model revealed an age related increased proportion of ever had penile vaginal sex where as age increased the proportion of sexual experience was increased. Respondents aged 20 years and older significantly more likely than those below 20 years to report that they had sexual experience. This is in line with reports from 2011 EDHS[22].

The proportion of ever had multiple sexual partners among those who had sexual intercourse was 42.7%. Similar comparable finding was noted in Bahir Dar city at private college students[29] and in Gonder[30]. However, high rate of multiple sex partners was reported in Wolaita University[31]. In contrast, a study in Haramaya[15] and Jimma University[4] reported lower rate of ever had multiple sexual partners. The difference could be due to difference in sample size, study population and comprehensive university based behavioural change interventions.

Engaging in risk behaviours such as Khat chewing, drinking alcohol, attending night clubs and watching porno videos were independently associated with likely hood of ever had sex and having multiple sexual partners. It is in line with study from Slovakia[32] and other universities in Ethiopia[4, 14–16]. This could be due to risk perception ability decreases with alcohol and khat consumption as a result students may not be capable of rational judgment and they also may not be able to predict the serious consequence of their action.

The frequency of unprotected sexual practice in this study (62%) was comparable with study done in Jimma University (57.6%)[4] and higher education of Cambodia[33]. However, it was higher than study from Medawolabu University (40.4%), Ethiopia[34]. Moreover, the level of consistently use of condom (38%) among sexually active students was lower than other studies, Ethiopia[15, 29, 34] that documented 48% - 81% of consistently condom use. This may be because of dynamicity of adolescent’s behaviour, difference in knowledge on risky sexual behaviours, reproductive health issues and skills of condom use.

According to this study, 7.4% of the sexually active students had sexual intercourse with commercial sex workers. This is lower than findings of other Universities in Ethiopia[4, 31, 34] where the rate of sex with commercial sex workers was 13.9% to 24.9%. This difference might be difference in awareness about mode of transmission and risk sexual behaviours among students in different Universities. Although attending night clubs was the only risk factor for engaging in sexual intercourse with commercial sex workers, Bahir Dar University has been started regulation mechanism that might limits the students from attending night clubs. The rules prevent students not to stay out of the campus during the night time.

In this study, the occurrence of exchanging sex for the sake of money was 4%. It is comparable with the cumulative proportion of other Universities of Ethiopia (4.4%)[4, 14, 15]. In contrast, it is lower than other study in Bahir Dar city[35] private college students and Addis Ababa where exchange sex among adolescents was 20.6%[36] and among university students was 14.5%[37]. Exchanging sex for money was significantly practiced in female than males.

In many societies young women have sexual relationship with men who are considerably older than them. This practice can contribute to the spread of HIV and other STIs because older men are more likely to have been exposed for those diseases. In this study, 21.5% of sexually active respondents had sex with older individuals. Similarly, according to EDHS study, over all 21% of women age 15–19 who had sexual intercourse had sex practice with a man ten or more years older than them and very few young men, <1% had sex with older women[22].

The proportion of watching porn videos in this study (65.4%) is comparable with other finding in Ethiopia (47.2%)[30]. However, our finding was significantly higher than studies done at Medawolabu (15.6%)[34] and Jimma Universities (32.4%)[4]. The highest proportion of watching porn videos detected among males and those whose age > 24 years old respondents. This might be associated with existence of sub cultural difference.

In this study, the proportion of attending night clubs is comparable with study in Bahir Dar city private college[29] and Jimma university students[4]. Male respondents were 2.2 times to attend night clubs than females. This might be associated with males feel more freedom and comfort to attend night clubs than females because of local cultural influence. Ethnical difference was also significantly associated with attending night clubs (Table 5). This might be associated with sub cultural difference and influence of the values and norms of the local community.

The major limitation of this study was the nature of cross sectional study which may not explain the temporal relationship between the outcome variable and some explanatory variables. The study topic by itself assesses personnel and sensitive issues related to sexuality which might have caused social desirability bias. Thus, the finding of this study should be interpreted with these limitations.

## Conclusions

The study revealed a more comprehensive understanding of the sexual behaviours of Bahir Dar University students. Risky sexual behaviours such as early age sex, having multiple sexual partners, unprotected sex, and sex with commercial sex workers are significantly practiced among students in Bahir Dar University. Substance use, attending night clubs and watching porno video were predictor factors for the existence of different sexual behaviours. Therefore, preventive intervention programmes should be strengthened, effectively implemented and monitored both in the earlier school and at the university level.

## Authors’ information

WML Assistant professor at College of Medicine and Health Sciences, Bahir Dar University in Medical Microbiology. BAB Associate professor of Medical microbiology, department head of Microbiology, Immunology and Parasitology at College of Medicine and Health Sciences, Bahir Dar University. MYM Assistant professor at College of Medicine and Health Sciences, Bahir Dar University in Medical Parasitology.
